# Removal of Plastics from Micron Size to Nanoscale Using Wood Filter

**DOI:** 10.3390/ma17061361

**Published:** 2024-03-16

**Authors:** Min Li, Gonggang Liu, Chongqing Wang, Shanshan Chang, Jinbo Hu

**Affiliations:** 1 Hunan Province Key Laboratory of Materials Surface & Interface Science and Technology, College of Materials Science and Engineering, Central South University of Forestry and Technology, Changsha 410004, China; 20221100242@csuft.edu.cn (M.L.); changelxy@hotmail.com (S.C.); hjb1999@hotmail.com (J.H.); 2 School of Chemical Engineering, Zhengzhou University, Zhengzhou 450001, China

**Keywords:** microplastics, nanoplastics, wood, PDDA, removal rate, size-exclusive interception, electrostatic interaction

## Abstract

Plastic pollution, particularly microplastic (MP) and nanoplastic (NP) pollution, has become a significant concern. This study explores the use of porous wood for filtration to remove MPs and NPs and investigates their removal mechanisms. Undecorated fir wood with a thickness of 4 mm achieves a 91% removal rate for model polystyrene (PS) MPs (2.6 μm) at a water flux of 198 L/m^2^h. However, its separation performance for NPs (255.8 and 50.9 nm) is poor. It also shows that fir wood (coniferous wood) has a higher PS removal rate than poplar wood (hard wood). With poly dimethyl diallyl ammonium chloride (PDDA) modification, both MPs and NPs are effectively removed, with NPs’ removal rate increasing from <10% to 90% for PDDA/wood. Characterization results reveal that size-exclusive interception dominates for micron-sized particles, and electrostatic interaction is crucial for nanosized particles. Additionally, intercepted NPs have been used as a strong binder for hot-pressed wood to remarkably enhance the mechanical properties of wood, suggesting a novel recycle utilization of discarded wood filters. Overall, this renewable wood material offers a simple solution for tackling MP/NP pollution.

## 1. Introduction

Plastics’ elimination from water is a key and emerging challenge, especially for the disposal of microplastics (particles smaller than 5 mm) and nanoplastics (particles smaller than 1 μm) [1,2,3]. Smaller particles of plastics enter the environment through various channels such as the degradation of existing plastic waste, which comes from plastic bottles, plastic bags, and other regularly used plastic items [4,5,6]. It is tough to ignore the problem of plastic pollution, which poses a great threat to oceanic life forms, birds, plants, and humans [7,8,9]. Studies have shown that nano- and microplastics can transfer through the food chain, potentially reaching humans who consume contaminated seafood [10]. Furthermore, nano- and microplastics have been found in various water bodies around the world, including rivers, lakes, and even tap water; water is identified as the perfect carrier media for plastic debris spread worldwide [11]. This highlights the widespread distribution of these particles and raises concerns about their potential effects on both aquatic life and human health. Therefore, to mitigate the risk of such pollutants, effective nano-/microplastic removal technologies from water need to be exploited urgently.

Currently, many technologies have been attempted to remove nano-/microplastics from water, such as coagulation, centrifugation, and membrane separation [12,13,14,15]. Among these remediation methods for MPs and NPs, membrane technologies are known for their low energy consumption, high removal efficiency, and space-efficient utilization [16,17,18]. Membrane materials are crucial to nano-/microplastics’ separating properties and scale application. Generally, polymeric membranes and ceramic membranes are widely used in the removal of nano-/microplastics from water [19,20,21,22]. In a study conducted by Xu et al. [23], both polyvinylidene fluoride (PVDF) and polyethersulfone (PES) membranes were utilized for the extraction of microplastics from lake water, achieving an impressive removal rate of 92%. However, polymeric membranes made from various polymers such as polyethylene, polypropylene, polysulfone, and PVDF also contribute to the secondary pollution problem when discarded, as they may form nano-/microplastics hazardous waste [24,25]. The research conducted by Kook et al. [26] illuminates the profound impact of ceramic membranes in capturing microplastics. Their findings not only underscore the efficacy of ceramic membranes but also shed light on their pivotal role in mitigating microplastic pollution, thereby contributing significantly to environmental conservation efforts. Nevertheless, ceramic membranes, though they are known for their high resistance to chemical and thermal degradation, making them suitable for separation application, have a higher cost in water treatment [27]. Additionally, appropriate pore structure design is also very important in obtaining high separation efficiency and flux simultaneously in the disposal of nano-/microplastic-containing water. On the other hand, the disposal of discarded membranes after contamination is also a nonnegligible issue.

As a kind of renewable and green biomass material, wood has been widely used for centuries in construction, furniture making, and energy production [28,29]. In recent years, wood has also been used to design high-flux membrane materials on account of its unique porous structure, decent mechanical and chemical durability, good modifiability, low cost, excellent hydrophilicity, and ease of processing, making it attractive in water treatment applications [30,31,32]. Notably, wood’s porous structure consists of numerous long and arrayed lumens interconnected by pits and ray cells, forming an oriented interpenetrating capillary network [33,34]. They provide a quick nutrition transmission channel in the growth direction, facilitating tree growth. Many studies have demonstrated that utilizing the pore structure of wood along its growth direction enables a highly efficient mass transfer in water treatment [35,36,37,38]. Especially in Hu’s research [39], the water treatment flux to degrade methylene blue (MB) in the wood membrane can reach up to 1 × 10^5^ L·m^−2^·h^−1^ with a high MB removal efficiency (>99.8%) concurrently, displaying high water treatment efficiency. In view of the above analysis, wood with an abundant and oriented microsized pore structure is an ideal green and renewable membrane filter. But so far, there has been no literature reporting on the application of wood in the disposal of nano-/microplastics.

Poly dimethyl diallyl ammonium chloride (PDDA) is a versatile chemical compound with diverse applications across various fields. Its wide-ranging utility spans water treatment, nanomaterial synthesis, biomedicine, electrochemistry, and surface modification [40,41,42]. PDDA is a positively charged polymer with excellent cationic properties [43]. This allows a strong electrostatic interaction with negatively charged plastic particles, facilitating efficient adsorption and removal. In addition, PDDA also demonstrates good chemical stability, ensuring the use stability and activity over extended periods in water treatment environments. Hence, it has been widely used as a reliable surface charge regulator for material surface modification.

**Figure 1:**
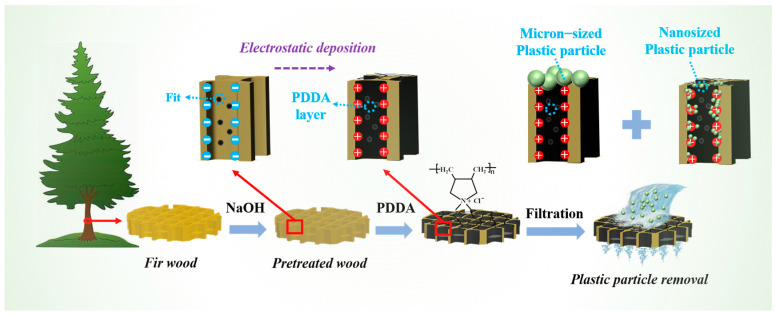
Synthesis routes for PDDA/wood and schematic of nano-/microplastic removal process by filtration.

In this work, wood and PDDA-modified wood have been used for MP and NP removal in the filtration process, and their removal mechanisms were further discussed. The effects of PDDA modification, wood species, and various thicknesses on the membrane filtration performance of MPs and NPs have been investigated. Meanwhile, the mechanical properties of hot-pressed wood samples with and without intercepted nanosized plastic particles have been tested to develop a novel recycling utilization of the discarded wood filter. Figure 1 shows the synthesis routes for PDDA/wood and a schematic of the nano-/microplastic removal process by PDDA/wood filtration.

## 2. Experimental

### 2.1. Materials and Reagents

The fir and poplar were used as raw materials, which were acquired via simple wood processing. They were cut into samples with a diameter of 25 mm and thicknesses of 2 mm and 4 mm. Sodium hydroxide (NaOH) and poly dimethyl diallyl ammonium chloride (PDDA) were purchased from Shanghai Titan Technology Co., Ltd. (Shanghai, China), and the commercial polystyrene beads (three types of non-functionalized PS beads with particle sizes of 50 ± 10 nm (PS50), 300 ± 20 nm (PS300), and 3 ± 0.2 µm (PS3000), respectively) were ordered from Ropes & Gray Technology Co., Ltd. (Wuxi, Jiangsu, China), and they were used directly.

### 2.2. Fabrication of Wood-Based Filter

The wood slices were immersed in 5 mol/L NaOH, heat-treated for 4 h at 60 °C, and washed with deionized (DI) water to remove gums and fatty acids, obtaining wood samples with good permeability. Then, they were put into a drying oven at 40 °C for 24 h. Next, 4% PDDA aqueous solution was prepared, and PDDA/wood was fabricated by suction filtration of 4% PDDA aqueous solution (40 mL) using the dried wood samples. Subsequently, the samples were soaked overnight in a 4% PDDA solution (100 mL), washed with deionized water, and then dried to obtain PDDA/wood. To study the effect of the PDDA loading method on the separation performance of nanoplastics (NPs), another method of PDDA/wood preparation adopted was ultrasonic loading in a 4% PDDA aqueous solution. In the ultrasonic loading process, the alkali-treated and dried wood is immersed in a 4% PDDA solution, where it absorbs PDDA through ultrasonic impregnation. After 1 h of ultrasonic treatment, the wood is filtered through a sand core funnel with deionized water and subsequently dried for future use.

### 2.3. Removal of Nano-/Microplastics from Water

PS beads dispersed in deionized water using ultrasound serve as the simulated plastic particle-containing solution. The absorbance of the PS solution (PS50, PS300) is then measured using a UV/Vis spectrophotometer and subsequently converted to concentration through a standard curve. Here, *C_0_* denotes the initial concentration. Following filtration through the wood membrane, the absorbance of the PS solution is measured, and the resulting value is recorded as *C_f_*. For PS3000, turbidity is assessed using a turbidity meter, and concentration is also determined by utilizing a standard curve. Here, *C_0_* signifies the initial concentration, while *C_f_* represents the concentration of the filtered solution. For the removal process, the developed wood-based membranes were placed in a sand core filtration apparatus, with an effective membrane area (*A_E_*) of 1.7 cm^2^, for the suction filtration tests. Each single suction filtration run contains 10 mL of PS-spiked solution (*V_f_*) with various particle sizes (50 nm, 300 nm, 3 µm). The operation time (*t_f_*) was counted from the start point to the time point where no additional permeate water drops were detected in two minutes of filtration. The removal and water flux were calculated using the following Equations (1) and (2), respectively:

(1) $$\mathrm{Removal}=\frac{\left(C_{0}-C_{f}\right)}{C_{0}}\times 100\%$$

(2) $$\mathrm{Water}\;\mathrm{flux}=\frac{V_{f}}{A_{E}{\;\times \;t}_{f}}$$

### 2.4. Characterization Analysis

#### 2.4.1. Scanning Electron Microscopy (SEM)

Surface morphology was examined using a scanning electron microscope (SEM, TescaMIRA3, TESCAN, Prague, Czech Republic) equipped with an EDX spectrometer for elemental mapping. The morphology of PS50, PS300, and PS3000 particles was observed, along with imaging of the wood-based membranes before and after the filtration process. Prior to analysis, all samples were coated with gold to ensure membrane and particle conductivity.

#### 2.4.2. Fourier Transform Infrared Spectroscopy (FTIR)

The chemical composition of fir and PDDA/wood was analyzed using a Fourier transform infrared spectrophotometer (FTIR spectrophotometer, Nicolet iS20, Waltham, MA, USA). Infrared spectra were acquired using a Thermo Scientific Nicolet spectrometer (Waltham, MA, USA) equipped with a KBr beam splitter. Spectra were recorded in the wavenumber range of 400–4000 cm^−1^ with a resolution of 4 cm^−1^.

#### 2.4.3. Zeta Potential Analyzer

The surface zeta potential (ζ-potential) of fir, PDDA/wood, PS50, PS300, and PS3000 was evaluated using a zeta potential analyzer (Nano ZS90, Malvern Zetasizer, Malvern, UK). Deionized water served as the dispersant for testing fir and PDDA/wood powders, with no ultrasonic treatment or pH adjustment applied during the analysis of fir and PDDA/wood. For the PS particle tests, deionized water was used as the dispersant, with ultrasonic treatment lasting 5 min.

#### 2.4.4. The Absorbance of PS50/PS300 and the Turbidity of PS3000 Solution

The absorbance of the PS solution (PS50, PS300) is measured by using a UV/Vis spectrophotometer (TU-1810PC, Shanghai Huyueming Co., Ltd., Shanghai, China). To ensure a comprehensive and accurate characterization, we employed a turbidity meter (SGZ-200BS, Shanghai Yuefeng Co., Ltd., Shanghai, China) to assess the turbidity of PS3000, as it was unresponsive to UV-Vis spectrophotometry [44]. It is crucial to meticulously calibrate the turbidity meter with deionized water and a standard calibration solution before conducting measurements. This step is fundamental in achieving dependable and precise turbidity readings.

#### 2.4.5. Optical Contact Angle Meter

The hydrophilicity of the wood samples was assessed using an optical contact angle meter (OCA15). Prior to measurement, the samples were dried to a constant weight. Rectangular shapes were cut from both fir and PDDA/wood. Deionized water served as the test solution, and the intravenous drip method was employed for testing. Data were recorded at 0.5 s intervals.

#### 2.4.6. Mechanical Testing Machine

The Young’s modulus and tensile strength of both the hot-pressed fir and the hot-pressed fir filtered with plastics were determined. This evaluation was conducted using an electronic universal material testing machine (HY0350, Hengyi Precision Instrument Co., Ltd., Shanghai, China). Before this, the samples undergo a series of preparation steps. After filtering PS50 through the wood sample, the sample was dried at 40 °C. Subsequently, the sample was subjected to the laboratory-designed hot press, which was heated to 140 °C under a pressure of 5 MPa for 30 min. Finally, the sample was allowed to cool to room temperature and then removed from the press for the mechanical test.

## 3. Results and Discussion

**Figure 2:**
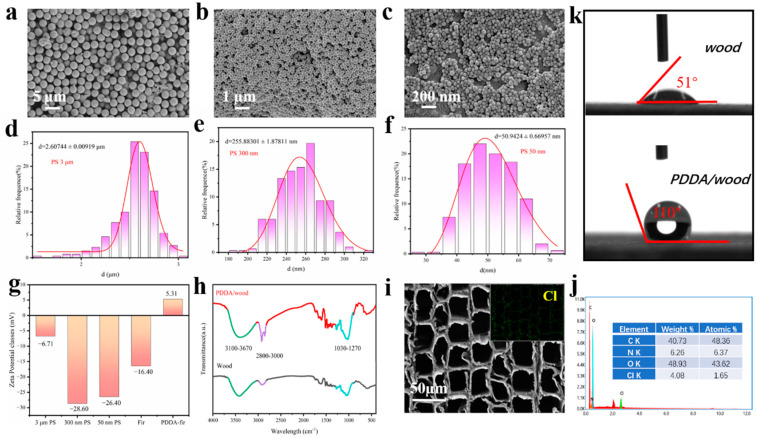
(**a**–**c**) SEM images of PS3000, PS300, PS50; (**d**–**f**) statistical analysis of plastic particle size from their SEM images; (**g**) zeta potential of PS3000, PS300, PS50, fir wood, and PDDA-modified wood; (**h**) FTIR analysis of wood and PDDA/wood; (**i**,**j**) SEM image and EDS analysis of PDDA/wood; (**k**) optical contact angle of wood and PDDA/wood.

First, model MPs and NPs including PS3000, PS300, and PS50 were characterized to further elucidate the removal mechanisms. SEM characterization and statistical analysis of the plastic particle sizes from their SEM images are shown in Figure 2a–f. SEM results show both PS MPs and NPs have a uniform particle size, and various PS samples display obvious size differences, which is beneficial for elucidating the removal mechanisms. Meanwhile, the results from the statistical analysis further confirm that the actual sizes of three PS particles (2.6 μm, 255.8 nm, 50.9 nm) are basically in accordance with the original design (3 μm, 300 nm, 50 nm). Meanwhile, to illustrate the role of electrostatic interaction in PS removal for wood-based samples, zeta potentials of PS3000, PS300, PS50, fir wood, and PDDA-modified wood have been provided, as shown in Figure 2g. The zeta potentials of various PS samples are −6.70, −28.60, and −26.40 mV, respectively, suggesting a negative surface charge of these MPs and NPs. Additionally, the surface charge of fir wood after PDDA modification was reversed from a negative value (−16.40 mV) to a positive value (+5.30 mV). This contributes to the successful modification of PDDA as a poly-cationic polyelectrolyte. As a consequence, the positively charged surface of PDDA/wood helps to adsorb the negatively charged polymer particles. In particular, when the size of the membrane pores significantly exceeds that of the polymer particles, electrostatic interactions emerge as pivotal factors in the separation process of plastic particles. The FTIR spectra of wood and PDDA/wood samples are shown in Figure 2h. For the PDDA/wood sample, the peaks at 2800 cm^−1^ to 3000 cm^−1^ are attributed to the quaternary ammonium groups of PDDA [45]. In addition, the SEM image and EDS spectrum of the PDDA/wood sample have been provided in Figure 2i,j. It was found that PDDA/wood maintains the porous array structure of the aligned lumens of fir wood. And the Cl element from PDDA is uniformly distributed over the whole surface of the wood. High mass content of N (6.26%) and Cl (4.08%) are found in a transverse section of the PDDA/wood surface. On the other hand, the surface water contact angle of the PDDA/wood also increased from 51° to 110° in Figure 2k, which may be due to the modified polymer chain structure on the wood’s surface. All of these results further confirm the successful PDDA modification.

**Figure 3:**
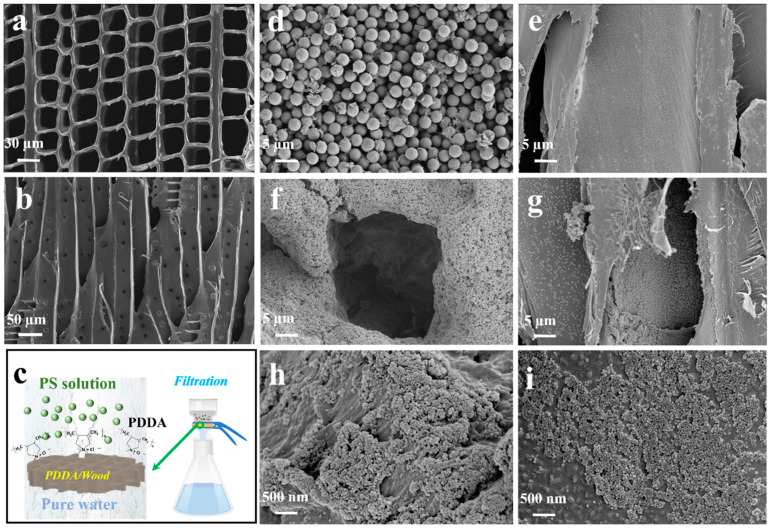
(**a**,**b**) SEM images in fiber direction and direction perpendicular to the fiber; (**c**) the membrane filtration process of PDDA/wood; SEM images of PDDA/wood after PS3000 filtration in (**d**) cross- and (**e**) interior sections; SEM images of PDDA/wood after PS300 filtration in (**f**) cross- and (**g**) interior sections; SEM images of PDDA/wood after PS50 filtration in (**h**) cross- and (**i**) interior sections.

The pore structure of fir wood samples is further illustrated from SEM images in the fiber direction and the direction perpendicular to the fiber, as shown in Figure 3a,b. Plenty of ordered, approximately rectangular micropores with a side length of about 20–30 μm are clearly observed along the transverse direction. It is derived from the cellular structure of a natural tree, which provides quick and effective passages for the transport of water and nutrition in the growth of living beings. Additionally, a large number of pits on the wood cell wall could be observed. These pits, about several microns in size, connected wood cell cavities to each other, constituting transversal fluid transport. As a consequence, these transmission channels are able to filter larger sizes of PS particles. After PDDA modification, the positively charged surface of the wood is capable of removing the negatively charged nanosized polymer particles from the huge cell wall surface area by electrostatic attraction. The membrane filtration process of PDDA/wood is shown in Figure 3c. To observe the membrane filtration result of various PS samples by PDDA/wood visually, SEM characterizations of PDDA/wood after PS3000, PS300, and PS50 filtration have been provided, respectively, as shown in Figure 3d–i. It could be found that PS3000 particles are piled on the surface of PDDA/wood, and its grid-like pores are hardly observed. Meanwhile, there are almost no PS3000 particles in the dissected fir wood cell surface. This has proven that PS particles with a larger size are directly rejected by the surface grid-like pore structure. For PS300 samples, the surface pore structure could be found, and plenty of PS300 particles are absorbed on the PDDA/wood surface. In addition, PS300 particles are also observed in the dissected fir wood cell surface, which means PS300 particles are able to enter into the interior of PDDA/wood. A similar result could be found for PS50 filtration with smaller-sized particles. The large interfacial area (estimated to be up to 6 × 10^4^ m^2^/m^3^) is capable of offering a huge adsorption area to reject nanoplastics [30].

**Figure 4:**
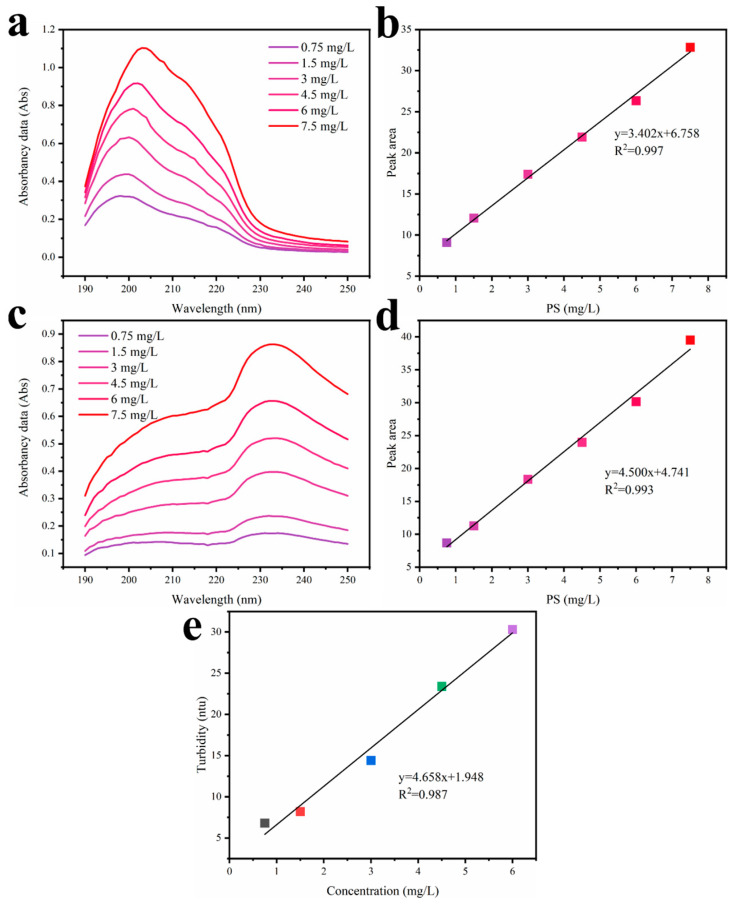
The concentrations and standard curves of (**a**,**b**) PS300 and (**c**,**d**) PS50 beads was analyzed using ultraviolet–visible (UV-vis) adsorption spectroscopy; (**e**) the standard curves of PS3000 concentration analyzed using a turbidity meter.

The removal rates and water flux of plastic particles of various sizes were collected and compared. Before determining the removal rates of PS3000, PS300, and PS50, the test method of PS concentration was studied. The concentration of PS300 and PS50 beads was analyzed using ultraviolet–visible (UV-vis) adsorption spectroscopy, and the standard curves were also made, as shown in Figure 4a–d. These figures indicate the concentrations of both PS300 and PS50 beads have a good linear relation with the light absorption intensity. For PS3000, its concentration was analyzed using a turbidity meter, and the standard curves were also made, as shown in Figure 4e. Similarly, it has a good linear relation. The results demonstrate the reliability of the PS concentration test.

**Figure 5:**
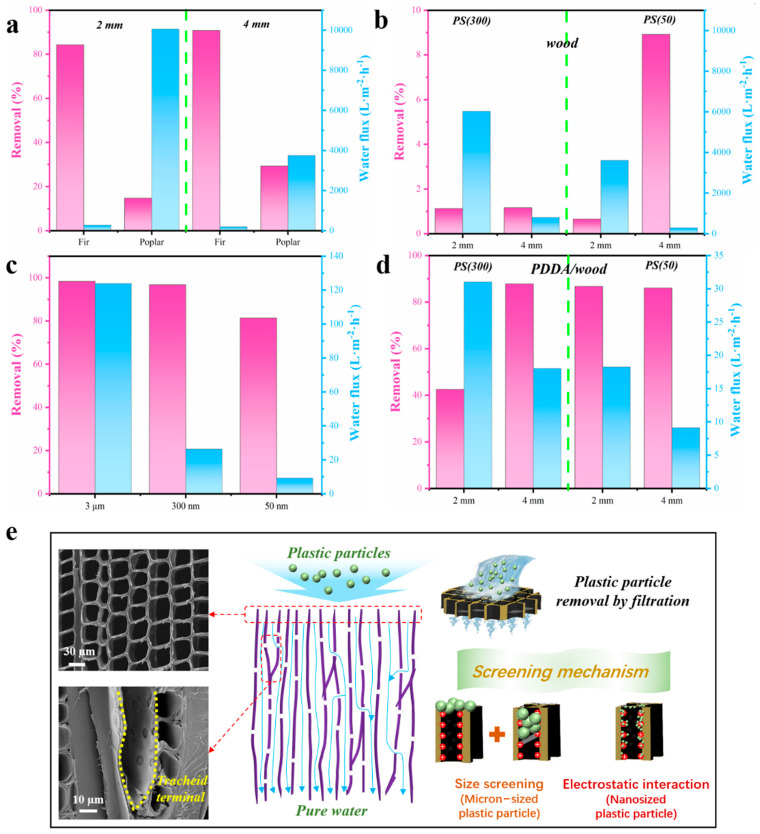
(**a**) The removal rates and water fluxes of microplastics (PS3000) by various wood samples with the thickness of 2 mm and 4 mm; (**b**) the removal rates and water fluxes of nanoplastics (PS300 and PS50) by fir wood with the thickness of 2 mm and 4 mm; (**c**) the removal rates and water fluxes of plastic particles with various size by PDDA/wood prepared by suction filtration; (**d**) the removal rates and water fluxes of nanoplastics (PS300 and PS50) by PDDA/wood with the thickness of 2 mm and 4 mm prepared by ultrasonic method; (**e**) schematic diagram of removal mechanism of microplastics (MPs) and nanoplastics (NPs) for PDDA/wood.

Figure 5a shows the removal rates and water fluxes of microplastic (PS3000) by various wood species with thicknesses of 2 mm and 4 mm. For PS3000 filtration, fir wood as a kind of coniferous wood shows a higher removal rate than poplar as a kind of hard wood. At the thickness of 4 mm, the removal rate of PS3000 for fir wood could reach 91%, much higher than that of poplar wood (29%). And an average water flux of 198 L/m^2^h could be obtained. However, the water flux of fir wood is lower than that of poplar wood. This is mainly due to the presence of vessels with a large size of about 80 μm in poplar wood [39], which are unable to reject PS3000 (average size is 2.6 μm). Additionally, it could be found that a larger thickness is beneficial for achieving a higher removal rate compared with wood samples with various thicknesses. The removal rates and water fluxes of nanoplastics (PS300 and PS50) by fir wood with a thickness of 2 mm and 4 mm are shown in Figure 5b. From the filtering result, undecorated fir wood has a low removal rate not exceeding 10%, both for PS300 and PS50 nanoplastics, and it has a similar trend with the increased thickness. The poor removal rates for nanoplastics are mainly because the micron-scale size of fir wood’s pore structure makes it difficult to effectively reject PS50 and PS300 solely by size sieving. Surprisingly, the removal rate of PS50 by fir wood at the thickness of 4 mm is higher than that of PS300. This is mainly because the pits enabling transversal fluid transport play an important role in water transmission at larger thicknesses, as the length of fir tracheids are several millimeters and adjacent tracheids are connected by these pits. As a consequence, PS50 with a smaller size enters into pits more easily and blocks the transmission path, leading to a higher removal rate. For comparison, PDDA/wood with a thickness of 2 mm displays much higher removal rates for both microplastics (MPs) and nanoplastics (NPs), as shown in Figure 5c. However, its water flux is unremarkable. Hence, another PDDA modification method has been studied in which the PDDA/wood was prepared by ultrasonic loading in 4% PDDA aqueous solution in Figure 5d. It could be found that the water fluxes of PDDA/wood with the thickness of 2 mm for PS300 and PS50 are increased obviously, which is mainly because suction filtration of PDDA loading in pressure can block pores with PDDA polymer molecules more easily compared with the ultrasonic method. Additionally, the effects of PDDA/wood thickness on the removal rates and water fluxes of nanoplastics (PS300 and PS50) are also studied. The result shows samples with a 4 mm thickness have a higher removal rate both for PS300 and PS50. According to the analysis from the SEM characterization and zeta potential results, a possible removal mechanism of microplastics (MPs) and nanoplastics (NPs) by wood-based membranes has been proposed, as shown in Figure 5e. Microplastics (MPs) and nanoplastics (NPs) enter aligned wood lumens through water. However, MPs with a larger size are directly rejected by the surface grid-like pore structure of PDDA/wood. For NPs (like PS50 and PS300), electrostatic interaction and size-exclusive interception have a combined effect. The huge capillary interfacial area of PDDA/wood could provide a positive charge to adsorb nanosized plastic particles, and pits are also used as screening channels at larger thicknesses. The removal mechanisms are determined as the following: (1) size-exclusive interception from internal pits and the tracheid terminal, as well as the external array pore structure, which dominates the removal for micron-sized plastic particles; (2) electrostatic interaction with the huge capillary interfacial area with positive charge caused by PDDA modification for nanosized plastic particles.

**Figure 6:**
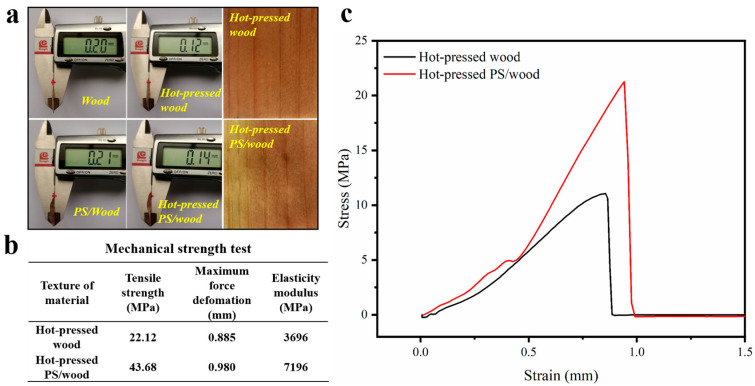
(**a**) The digital photos of wood and PS/wood before and after hot press; (**b**) the comparison of the mechanical strength of hot-pressed wood and PS/wood; (**c**) the stress–strain curve of hot-pressed wood and PS/wood.

On the other hand, to realize the maximum utilization of resources, a novel recycling utilization of discarded wood filters after NP filtration has been put forward. Intercepted nanosized plastic particles have been used as strong binders for hot-pressed wood in order to enhance the mechanical properties of the wood. The results are shown in Figure 6. After hot press at 5 MPa and 140 °C, both wood and PS/wood showed a large compression in thickness. Meanwhile, the tensile strength and elasticity modulus of PS/wood increased from 22.12 and 3696 MPa to 43.68 and 7196 MPa, respectively. 

## 4. Conclusions

In this work, wood with a high porosity and unique transport channels has been successfully used for MP and NP removal by a filtration process for the first time. PDDA has been successfully adopted to modify the wood and adjust its surface charge. Undecorated fir wood displays a decent PS MP removal rate with high water flux, resulting in poor separation performance for NPs. Fir wood as a kind of coniferous wood has a higher PS removal rate than poplar wood (hard wood). Meanwhile, PDDA-modified wood has an excellent rejection performance, both for MP and NP removal, with decent water fluxes. Removal mechanisms have been proposed, which include size-exclusive interception from the wood’s pore structure for MPs and electrostatic interaction from PDDA modification for NPs. Additionally, a novel recycling utilization of discarded wood filters after NP filtration has been put forward, which demonstrates that rejected NPs in wood membranes could be used as strong binders for hot-pressed wood to remarkably enhance the mechanical properties of the wood. In summary, our work demonstrates that the renewable wood material has been successfully used to remove MPs/NPs and gives a novel recycling utilization for discarded wood filters, which provides a promising way to tackle MP/NP pollution.

## Data Availability

Data are contained within the article.
